# No Associations between Dairy Intake and Markers of Gastrointestinal Inflammation in Healthy Adult Cohort

**DOI:** 10.3390/nu15163504

**Published:** 2023-08-08

**Authors:** Yasmine Y. Bouzid, Elizabeth L. Chin, Sarah S. Spearman, Zeynep Alkan, Charles B. Stephensen, Danielle G. Lemay

**Affiliations:** 1 USDA ARS Western Human Nutrition Research Center, Davis, CA 95616, USA; 2 Department of Nutrition, University of California, Davis, CA 95616, USA

**Keywords:** dairy intake, gastrointestinal inflammation

## Abstract

Dairy products are a good source of essential nutrients and past reviews have shown associations of dairy consumption with decreased systemic inflammation. Links between dairy intake and gastrointestinal (GI) inflammation are under-investigated. Therefore, we examined associations between reported dairy intake and markers of GI inflammation in healthy adults in a cross-sectional observational study, hypothesizing a negative association with yogurt intake, suggesting a protective effect, and no associations with total dairy, fluid milk, and cheese intake. Participants completed 24-h dietary recalls and a food frequency questionnaire (FFQ) to assess recent and habitual intake, respectively. Those who also provided a stool sample (*n* = 295), and plasma sample (*n* = 348) were included in analysis. Inflammation markers from stool, including calprotectin, neopterin, and myeloperoxidase, were measured along with LPS-binding protein (LBP) from plasma. Regression models tested associations between dairy intake variables and inflammation markers with covariates: age, sex, and body mass index (BMI). As yogurt is episodically consumed, we examined differences in inflammation levels between consumers (>0 cup equivalents/day reported in recalls) and non-consumers. We found no significant associations between dairy intake and markers of GI inflammation. In this cohort of healthy adults, dairy intake was not associated with GI inflammation.

## 1. Introduction

The Dietary Guidelines for Americans recommend three servings of dairy each day as a source of essential nutrients, especially for under-consumed nutrients of concern such as calcium, potassium, and vitamin D [1]. Dairy consumption is known to be beneficial for bone health, to reduce the risk of cardiovascular disease and diabetes, and is associated with lower mortality [2,3,4,5]. Systematic reviews of randomized controlled trials report a neutral to positive, anti-inflammatory effect of dairy intake on biomarkers of inflammation [6,7,8,9,10,11]. Individuals with commonly experienced gastrointestinal symptoms such as abdominal pain, bloating, or diarrhea often avoid dairy on their own or are advised to do so by their health practitioners [12]. However, the effect of dairy products on gastrointestinal health in adults is largely understudied.

Very few studies of fecal markers of gastrointestinal inflammation in response to dairy products have been conducted with healthy adults. Fecal calprotectin, a clinical diagnostic for gastrointestinal inflammation, was measured in three studies: an intervention with whipping cream [13], a comparison of A1 and A2 milk [14], and a tolerance study for casein glycomacropeptide [15]. None of these studies have relevance for the Dietary Guidelines which recommend low-fat milk, cheese, and yogurt (but not whipping cream, ice cream, etc.). The association between short- or long-term recommended dairy intake and fecal calprotectin levels in healthy people remains unknown.

The healthy gastrointestinal tract absorbs nutrients while maintaining a protective barrier between gut bacteria and the bloodstream. Even fewer studies have investigated the effects of dairy intake with respect to gastrointestinal barrier function in healthy individuals. In an intervention study with healthy men, whipping cream intake resulted in no significant change in gut permeability as directly measured with non-metabolizable sugars [13]. Gastrointestinal barrier function is often indirectly measured by quantitating the abundance in plasma of lipopolysaccharide-binding protein (LBP), which increases when lipopolysaccharide, a component of gram-negative bacteria, bypasses the GI tract and enters the blood stream. A study of yogurt consumption compared with a soy control had ambiguous findings: individual markers of endotoxin exposure, such as LBP, did not change, but the ratio of the markers improved [16]. In vitro experiments suggest that dairy and dairy-derived products can improve intestinal barrier function [17,18].

A systematic review of yogurt and/or fermented dairy consumption and health identified associations of fermented dairy intake with improvements in GI symptoms, diarrhea, and constipation, as well as a causal relationship with lactose digestion [19]. Studies of yogurt intervention in patients with irritable bowel syndrome show mixed results with some improving symptoms relative to placebo [20,21], but no worsening symptoms [22,23,24]. We, therefore, hypothesized that yogurt consumption would be negatively associated with markers of GI inflammation and intestinal permeability in healthy adults.

Given the paucity of studies of dairy and gastrointestinal status in healthy adults, the objective of the current study was to examine the relationship of dairy intake with fecal markers of gastrointestinal inflammation and with plasma LBP in a multi-ethnic cohort of normal to moderately obese men and women, ages 18 to 65 years, for whom both recent and habitual dietary intake was assessed. Gastrointestinal inflammation was measured using fecal calprotectin, fecal myeloperoxidase, and fecal neopterin. While fecal calprotectin is a generic and commonly used clinical measure, fecal myeloperoxidase specifically increases with the involvement of neutrophils while fecal neopterin increases with the involvement of macrophages.

## 2. Materials and Methods

### 2.1. Participants

Study participants were healthy adults aged 18–65 years, with a BMI (kg/m^2^) of 18.5–45.0 (normal to obese), living near Davis, California. Participants were recruited in the cross-sectional USDA Nutritional Phenotyping Study as stratified by 18 categories defined by age, sex, and BMI to obtain a diverse sample population (NCT02367287) [25]. The purpose of the study was to characterize immunologic and physiologic phenotypes of healthy adults to identify factors that may be intervention targets to improve metabolic flexibility. Primary hypotheses included that higher diet quality would be associated with lower GI inflammation. Volunteers were excluded if they had been diagnosed with a chronic disease or if their blood pressure readings indicated hypertension at either of the two visits. A total of 393 participants were enrolled in the study, and 348 with complete dietary data, fasting plasma, and stool samples were included in the analyses.

### 2.2. Dietary Intake Assessment

Recent dietary intake in the form of three 24-h recalls was collected and analyzed using the Automated Self-Administered 24-h (ASA24) Dietary Assessment Tool, version (2016), developed by the National Cancer Institute, Bethesda, MD, USA (https://epi.grants.cancer.gov/asa24, accessed on 4 December 2020). Manual data cleaning was previously described [26]. The 24-h recalls were conducted in the 10-day period prior to the stool collection. There was a training recall (with staff present to assist the participant) followed by three prompts for at-home recalls, two weekdays and one weekend day. Total Energy Expenditure (TEE) was independently calculated using resting metabolic rate (measured using a metabolic cart) and physical activity levels (measured over the 10-days with an accelerometer). Calories reported via the training recall were lower, on average, than reported in the at-home recalls. Calories reported in the at-home recalls were more highly correlated with calculated TEE. We, therefore, used only the at-home recalls for the assessment of dairy intake. Habitual dietary intake was completed using the 2014 Block food frequency questionnaire (FFQ) with which participants were asked to report consumption of foods over the previous 12 months.

### 2.3. Stool Collection

Stool collection and processing procedures were described previously [27,28]. Briefly, volunteers collected a single stool sample at home, which was stored on ice immediately and brought to the Western Human Nutrition Research Center (WHNRC) as soon as possible. Fecal samples were homogenized, flash frozen and aliquoted by a technician, and stored at −70 °C until analyses.

### 2.4. Plasma Collection

Plasma was processed from fasting blood collected in either EDTA or heparin tubes immediately after the blood draw [28]. Plasma aliquots were stored at −80 °C until use.

### 2.5. Quantification of Gut Inflammation Markers

We measured the abundance of fecal calprotectin, myeloperoxidase, and neopterin via ELISA. We also used ELISA to measure the abundance of LPS-binding protein (LBP) in plasma collected at fasting.

### 2.6. Fecal Calprotectin and MPO

Calprotectin (Immundiagnostik, Bensheim, Germany; catalog [cat] number K6927) and MPO (Immundiagnostik; cat number KR6630) enzyme-linked immunosorbent assays (ELISAs) were used per kit instructions to analyze frozen homogenized stool samples, which were thawed slowly prior to extraction with the IDK Extract Stool Sample Preparation System (Immundiagnostik) as described in detail before [28].

### 2.7. Fecal Neopterin

Stool aliquots were extracted into a saline solution as published previously [29] and fecal neopterin was quantified from the extracts with ELISAs (B·R·A·H·M·S/ThermoFisher, Hennigsdorf, Germany; cat number 14-HD-99.1).

### 2.8. Plasma Lipopolysaccharide-Binding Protein (LBP)

LBP was quantified from clarified and 800-fold diluted fasting heparin plasma with ELISAs (Abnova, Taipei City, Taiwan; cat number KA0448) as described elsewhere [28].

### 2.9. Statistical Methods

R was used for statistical analysis and visualizations. Linear regression was used to examine associations between total dairy, fluid milk, cheese, and yogurt intake and stool calprotectin, myeloperoxidase, neopterin, and plasma LBP. Distributions for fecal calprotectin, and fecal myeloperoxidase were transformed with ln(x + 3) and ln(x + 1) transformations, respectively. Fecal neopterin was transformed using the Box Cox transformation. Plasma LBP distribution was transformed with an ln(x + 1) transformation. Age, sex, and BMI were included in the models to account for covariates when examining associations between dairy intake variables and markers of GI inflammation. Since yogurt is episodically consumed, the distributions for recent intake were zero-inflated. Therefore, we characterized yogurt consumers as those with any amount reported in averages of their 24-h recalls and ran similar regression models. Distributions for markers of GI inflammation were transformed using suggestions from the BestNormalize package when assessing associations with yogurt consumption: calprotectin (orderNorm), myeloperoxidase (Box Cox), neopterin (orderNorm), and LBP remained with the original ln(x + 1) transformation as residuals were normal when checked by Shapiro test. Student’s t-tests were also used to test differences in mean levels of GI inflammation markers between recent yogurt consumers and non-consumers.

## 3. Results

### 3.1. Participant Characteristics

Of the 348 participants included in this study, 164 were male and 184 were female. Mean age was 40.51 ± 13.7 years with a range of 18 to 66 years. Mean BMI was 27.28 ± 4.9 kg/m^2^ with a range of 18.04 to 43.87 kg/m^2^.

Participants reported recent total dairy intake, as an average across 24 h recalls as 1.60 ± 1.05 cup equivalents per day, range 0 to 6.71. They reported habitual consumption of 1.48 ± 1.09 cup equivalents of total dairy per day with a range of 0.21 to 8.06 (per the FFQ). As some dairy intake is aggregated from mixed dishes (e.g., scrambled eggs) in which a consumer may not have consumed dairy (e.g., scrambled eggs made without milk), some amount of dairy consumption may be incidental; if one conservatively (to minimize false positives at the expense of false negatives) defines those who consume more than 0.25 cups/day as a dairy consumer, then at least 92% of participants in this cohort would be defined as dairy consumers. Recent fluid milk consumption was 0.56 ± 0.65 cup equivalents per day, range 0 to 5.73. Habitual fluid milk consumption was 0.64 ± 0.72 cup equivalents per day, range 0.05 to 5.20. Recent cheese intake was 0.84 ± 0.79 cup equivalents per day, range 0 to 4.60. Habitual cheese intake was 0.84 ± 0.58 cup equivalents per day, range 0.09 to 4.00. Recent yogurt intake was 0.13 ± 0.25 cup equivalents per day, range 0 to 1.84. 57% of participants reported no recent yogurt intake. Habitual yogurt intake was 0.19 ± 0.25 cup equivalents per day, range 0 to 2.01. As yogurt is an episodically consumed food, yogurt consumers were defined as those who consumed more than 0 cup equivalents per day.

Mean calprotectin was 65.09 ± 136.43 μg/g with a range from 0 to 1878.79. Mean myeloperoxidase was 606.48 ± 1619.45 ng/g with a range from 13.75 to 21,668.50. Mean neopterin was 20.19 ± 27.98 ng/g with a range from 4.33 to 228.71. Mean LBP was 10.65 ± 6.00 μg/mL with range 1.00 to 38.27.

### 3.2. Association of Dairy Intake with Fecal Markers of GI Inflammation

**Figure 1:**
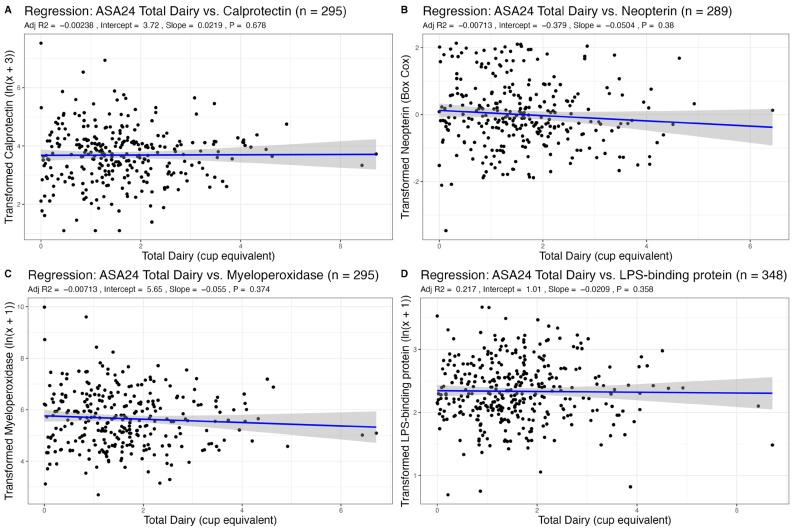
Association of recent total dairy intake (cup equivalent per day) with markers of GI inflammation adjusted for sex, age, and BMI.

**Figure 2:**
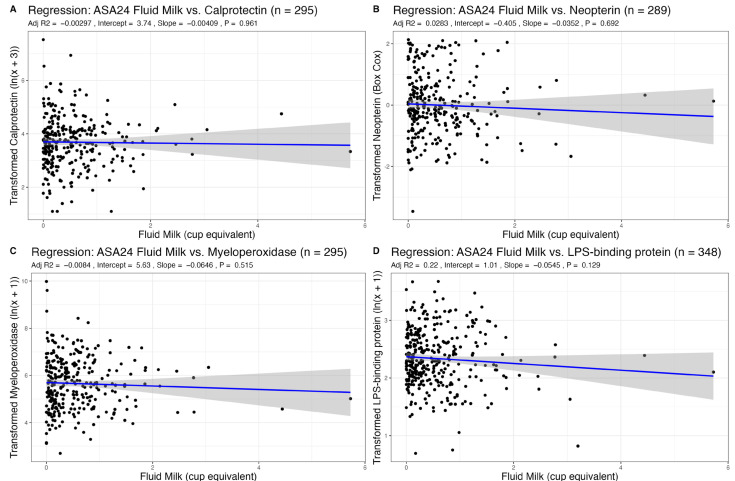
Association of recent fluid milk intake (cup equivalents per day) with markers of GI Inflammation adjusted for sex, age, and BMI.

**Figure 3:**
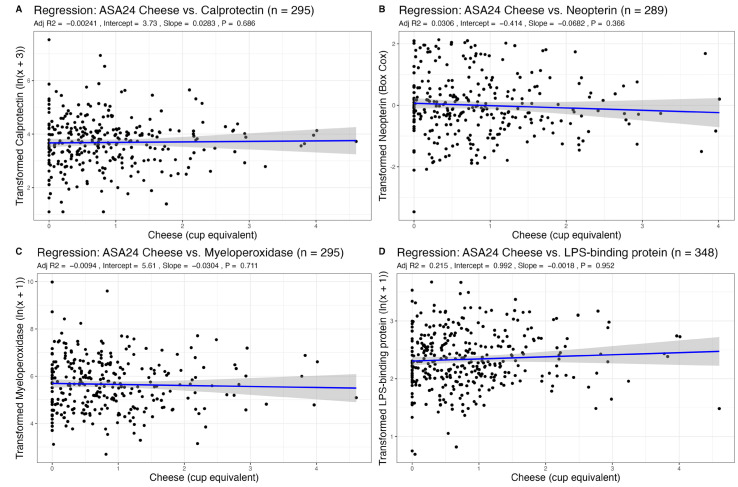
Association of recent cheese intake (cup equivalents per day) with markers of GI inflammation after adjustment for sex, age, and BMI.

Given that age, sex, or BMI may influence inflammation, we adjusted all analyses for these three covariates. We found no associations between recent intake of total dairy, fluid milk, or cheese, as measured via ASA24 recalls, with any fecal markers of inflammation (Table 1, Table 2 and Table 3, Figure 1, Figure 2 and Figure 3). There was also no association between habitual intake, as measured with an FFQ, of total dairy, fluid milk, or cheese with any fecal markers (Supplementary Tables S1–S3, Supplementary Figures S1–S3).

**Figure 4:**
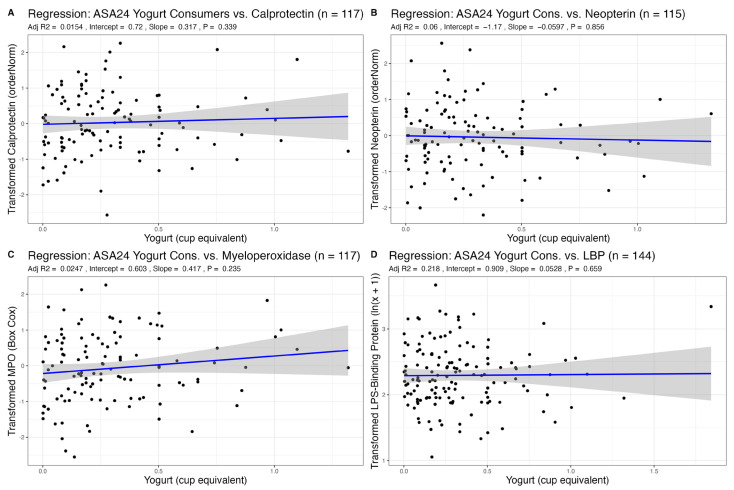
Association of recent yogurt intake (consumers only, >0 cup eq.) with markers of GI inflammation after adjustment for sex, age, and BMI.

Yogurt intake was a zero-inflated variable, particularly for ASA24 recalls. Therefore, we stratified subjects as non-consumers and consumers (>0 cup equivalents yogurt reported in their averaged recalls). We found no significant differences in fecal markers of inflammation between non-consumers and consumers of yogurt. When analyzing relationships only among consumers of yogurt, we found no association between the amount of yogurt recently consumed and markers of GI inflammation (Table 4, Figure 4). Using the FFQs, there was also no relationship between habitual consumption of yogurt and GI inflammation (Supplementary Table S4, Figure S4).

### 3.3. Association of Dairy Intake with Plasma LBP, a Marker of Endotoxin Exposure

**Figure 5:**
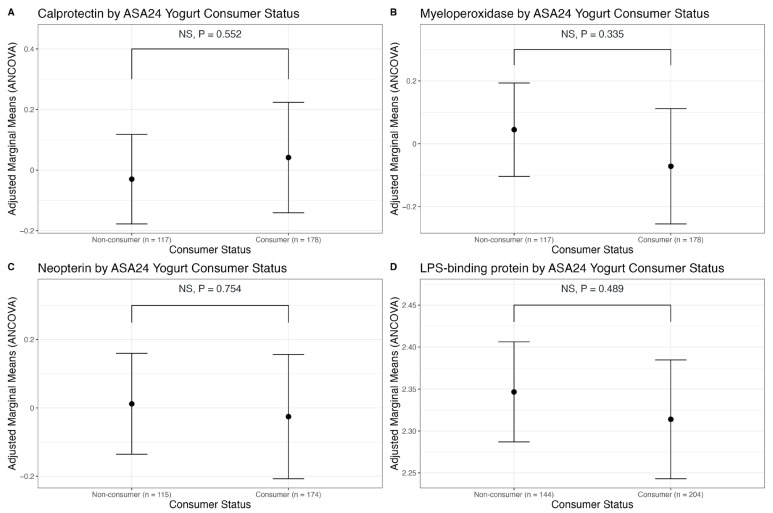
Points show adjusted marginal means from ANCOVA analysis for calprotectin (**A**), neopterin (**B**), myeloperoxidase (**C**), and LBP (**D**) based on consumer status. Error bars represent 95% confidence intervals. NS indicates *p*-values not significant.

The distribution for plasma LPS-binding protein (LBP), an indirect marker of endotoxin exposure, was transformed with an ln(x + 1) transformation. We found no associations between total dairy, fluid milk, cheese, and yogurt intake from ASA24 recalls (a measure of recent dietary intake) with plasma LBP (Table 1, Table 2, Table 3 and Table 4, Figure 1, Figure 2, Figure 3 and Figure 4). Using FFQ data (as a measure of habitual intake), we also found no significant association of dairy consumption with LBP levels (Supplementary Tables S1–S4, Supplementary Figures S1–S4). We also found no significant differences in plasma LBP between non-consumers and consumers of yogurt. There were no significant differences between mean GI inflammation markers by *t*-test (Figure 5).

## 4. Discussion

Previous studies have examined the association of dairy intake with systemic inflammation and found no or beneficial effects of dairy consumption (reviewed in [10]). An analysis of data from 35,352 postmenopausal women in the Women’s Health Initiative demonstrated that higher total dairy intake, cheese, and yogurt were associated with lower concentrations of C-reactive protein [30]. However, as large national surveys do not measure gastrointestinal inflammation, this has been a gap in the scientific literature. In our cohort of 348 multi-ethnic U.S. adults, we found no significant associations between dairy intake, fluid milk intake, cheese intake, or yogurt intake with fecal markers of gastrointestinal inflammation. We also found no association between dairy intake, fluid milk intake, cheese intake, or yogurt intake with plasma LBP, an indirect measure of gastrointestinal permeability.

Some adults avoid dairy consumption due to real or perceived lactose intolerance. We previously showed that the multi-ethnic participants in our cohort included more than 40% of participants with lactase non-persistent genotypes [31]. Despite the high incidence of genetic lactose intolerance in this cohort, we found no association of total dairy, fluid milk, or cheese intake with gastrointestinal inflammation.

Beta-casein, an abundant protein in milk, appears in different forms, such as A1 and A2, depending on the genetic variants present in the cow’s genome [32]. A randomized crossover double-blinded trial found that A2 milk reduced digestive symptoms in 600 Chinese adults [33]. However, a randomized crossover blinded clinical trial of 40 women in New Zealand showed that while lactose-intolerant participants experienced reduced symptoms with A2 milk, dairy tolerant participants had increased diarrhea [34]. The impact of A2 milk, relative to regular milk, which contains both A1 and A2 protein, remains controversial with more studies needed. In the current study, as A2 milk was not generally available in the U.S. during most of the years that participants were enrolled (2015–2019), it is a reasonable assumption that all or most of the fluid milk consumed by participants was not A2 milk. Nevertheless, we saw no association of fluid milk intake with GI inflammation.

Fermented dairy products have been shown to improve stool frequency or consistency in patients with constipation in small clinical trials [35,36]. In the EPIC-Italy cohort (*n* > 45,000) adults, yogurt consumption was association with reduced colon cancer risk [37] and it is known that chronic GI inflammation increases the risk of developing colon cancer [38]. Interventions with fermented milk products, which contain lactic acid bacteria (LAB), have been shown to attenuate GI inflammation in a mouse model of colitis though various mechanisms such as altering the gut microbiome to reduce colitogenic microbes [39], reducing production of Th1-type cytokines [40], reducing IL-6 by a polysaccharide peptidoglycan component of LAB [41], reducing IL-6 and TNF-α expression by fatty acids produced during LAB fermentation of milk [42], and activation of epidermal growth factor receptor on intestinal epithelial cells [43]. Therefore, we had hypothesized that increased yogurt consumption would be associated with a decrease in GI inflammation. However, we found no association between yogurt consumption and GI inflammation in healthy adults. It is possible that participants in our study may not have consumed enough yogurt with 57% of the cohort reporting no yogurt consumption in their 24 h recalls and with a median of 0.25 cups/day even among consumers.

The effect of yogurt consumption on intestinal permeability is not well-studied in humans, likely due to the invasiveness of such study. In a double-blind controlled trial, participants who were to undergo endoscopy and treated with low-dose aspirin for one month were randomized to consume either yogurt (220 mL/day) or placebo daily [44]. The patients consuming yogurt had fewer mucosal breaks and improvement in GI symptoms. In our study, we found no association between yogurt intake and plasma LBP, an indirect measure of gastrointestinal permeability, but our participants did not undergo a challenge such as low-dose aspirin.

A major limitation of our study is its observational nature. However, observational analyses are low-cost first steps prior to designing an intervention study and no previous observational study with GI endpoints had been conducted for dairy consumption. Another limitation is the exclusion of participants with GI disease. We, therefore, cannot infer an association beyond healthy people. However, as we did find associations of our GI endpoints with age, sex, and BMI in this cohort, the negative findings are not due to all participants having unremarkable GI outcomes. LBP was elevated in obese individuals (Table 1, Table 2, Table 3 and Table 4) and both age and sex were significant in some models of fecal GI markers (Table 4) with older individuals and females associated with higher inflammation, compared with those younger than 50 years and males, respectively. Another limitation is generalizability around the world as recommendations for dairy intake vary internationally with some countries grouping dairy under protein foods instead of a separate food group [45].

In summary, we found no association of dairy intake of any type with GI inflammation or with GI permeability in a multi-ethnic healthy U.S. cohort of adults who were heterogenous for lactose intolerance. Future studies are warranted, particularly for interventions with daily doses of yogurt to define effects on GI inflammation and/or GI permeability, perhaps with older (> 50 y), obese females, and incorporating a gastrointestinal challenge.

## Figures and Tables

**Table 1:** Results from linear regression between total dairy intake from 24-h recall (ASA24) and markers of GI inflammation adjusted for age, sex, and BMI. Results from linear regression between age, sex, or BMI vs. GI inflammation markers are also shown.

	Transformed Calprotectin		Transformed Myeloperoxidase		Transformed Neopterin		Transformed LPS-Binding Protein	
Predictors	Estimates (95% CI)	*p*-Value	Estimates (95% CI)	*p*-Value	Estimates (95% CI)	*p*-Value	Estimates (95% CI)	*p*-Value
Total Dairy	0.02 (−0.08–0.13)	0.678	−0.06 (−0.18–0.07)	0.374	−0.05 (−0.16–0.06)	0.380	−0.02 (−0.07–0.02)	0.358
Age	−0.01 (−0.01–0.00)	0.215	0.00 (−0.01–0.01)	0.860	−0.00 (−0.01–0.00)	0.353	0.00 (−0.00–0.01)	0.195
Sex	0.15 (−0.07–0.38)	0.175	0.11 (−0.15–0.37)	0.414	0.34 (0.10–0.57)	**0.005**	0.13 (0.04–0.23)	**0.005**
BMI	0.00 (−0.02–0.02)	0.846	0.00 (−0.03–0.03)	0.993	0.02 (−0.01–0.04)	0.166	0.04 (0.03–0.05)	**<0.001**
R^2^/R^2^ adjusted	0.011/−0.002		0.007/−0.007		0.044/0.030		0.226/0.217	

Bold indicates statistically significant *p*-values (alpha = 0.05).

**Table 2:** Results from linear regression between fluid milk from 24-h recall (ASA24) and markers of GI inflammation adjusted for age, sex, and BMI. Results from linear regression between age, sex, or BMI vs. GI inflammation markers are also shown.

	Transformed Calprotectin		Transformed Myeloperoxidase		Transformed Neopterin		Transformed LPS-Binding Protein	
Predictors	Estimates (95% CI)	*p*-Value	Estimates (95% CI)	*p*-Value	Estimates (95% CI)	*p*-Value	Estimates (95% CI)	*p*-Value
Fluid Milk	−0.00 (−0.17–0.16)	0.961	−0.06 (−0.26–0.13)	0.515	−0.04 (−0.21–0.14)	0.692	−0.05 (−0.12–0.02)	0.129
Age	−0.00 (−0.01–0.00)	0.226	0.00 (−0.01–0.01)	0.860	−0.00 (−0.01–0.00)	0.338	0.00 (−0.00–0.01)	0.169
Sex	0.14 (−0.08–0.36)	0.199	0.13 (−0.13–0.38)	0.332	0.35 (0.12–0.58)	**0.003**	0.13 (0.04–0.23)	**0.004**
BMI	0.00 (−0.02–0.02)	0.799	−0.00 (−0.03–0.02)	0.914	0.02 (−0.01–0.04)	0.202	0.04 (0.03–0.05)	**<0.001**
R^2^/R^2^ adjusted	0.011/−0.003		0.005/−0.008		0.042/0.028		0.229/0.220	

Bold indicates statistically significant *p*-values (alpha = 0.05).

**Table 3:** Results from linear regression between cheese intake from ASA24 and markers of GI inflammation adjusted for age, sex, and BMI. Results from linear regression between age, sex, or BMI vs. GI inflammation markers are also shown.

	Transformed Calprotectin		Transformed Myeloperoxidase		Transformed Neopterin		Transformed LPS-Binding Protein	
Predictors	Estimates (95% CI)	*p*-Value	Estimates (95% CI)	*p*-Value	Estimates (95% CI)	*p*-Value	Estimates (95% CI)	*p*-Value
Cheese	0.03 (−0.11–0.17)	0.686	−0.03 (−0.19–0.13)	0.711	−0.07 (−0.22–0.08)	0.366	−0.00 (−0.06–0.06)	0.952
Age	−0.00 (−0.01–0.00)	0.227	0.00 (−0.01–0.01)	0.908	−0.00 (−0.01–0.00)	0.312	0.00 (−0.00–0.01)	0.216
Sex	0.15 (−0.07–0.37)	0.178	0.13 (−0.13–0.39)	0.333	0.34 (0.11–0.57)	**0.004**	0.14 (0.05–0.23)	**0.003**
BMI	0.00 (−0.02–0.02)	0.869	−0.00 (−0.03–0.03)	0.972	0.02 (−0.01–0.04)	0.151	0.04 (0.03–0.05)	**<0.001**
R^2^/R^2^ adjusted	0.011/−0.002		0.004/−0.009		0.044/0.031		0.224/0.215	

Bold indicates statistically significant *p*-values (alpha = 0.05).

**Table 4:** Results from linear regression between yogurt from ASA24 (consumers only) and markers of GI inflammation adjusted for age, sex, and BMI. Results from linear regression between age, sex, or BMI vs. GI inflammation markers are also shown.

	Transformed Calprotectin		Transformed Myeloperoxidase		Transformed Neopterin		Transformed LPS-Binding Protein	
Predictors	Estimates (95% CI)	*p*-Value	Estimates (95% CI)	*p*-Value	Estimates (95% CI)	*p*-Value	Estimates (95% CI)	*p*-Value
Yogurt	0.32 (−0.34–0.97)	0.339	0.42 (−0.27–1.11)	0.235	−0.06 (−0.71–0.59)	0.856	0.05 (−0.18–0.29)	0.659
Age	−0.01 (−0.03–−0.00)	**0.026**	0.00 (−0.01–0.02)	0.711	0.01 (−0.00–0.02)	0.155	0.00 (−0.00–0.01)	0.120
Sex	0.10 (−0.24–0.43)	0.570	0.19 (−0.16–0.55)	0.283	0.46 (0.12–0.79)	**0.008**	0.18 (0.04–0.31)	**0.011**
BMI	−0.01 (−0.05–0.03)	0.622	−0.04 (−0.08–0.00)	0.061	0.02 −0.02–0.06)	0.278	0.04 (0.03–0.06)	**<0.001**
R^2^/R^2^ adjusted	0.049/0.015		0.058/0.025		0.093/0.060		0.240/0.218	

Bold indicates statistically significant *p*-values (alpha = 0.05).

## Data Availability

Not applicable.

## Supplementary Materials

The following supporting information can be downloaded at: https://www.mdpi.com/article/10.3390/nu15163504/s1, Table S1: Results from linear regression between total dairy intake (cup equivalents per day) from food frequency questionnaire (FFQ) and markers of GI inflammation adjusted for age, sex, and BMI; Figure S1: Association of habitual total dairy intake with markers of GI inflammation adjusted for sex, age, BMI; Table S2: Results from linear regression between fluid milk intake (cup equivalents per day) from FFQ and markers of GI inflammation adjusted for age, sex, and BMI; Figure S2: Association of habitual fluid milk intake with markers of GI inflammation after adjustment for sex, age, BMI; Table S3: Results from linear regression between cheese intake (cup equivalents per day) from FFQ and markers of GI inflammation adjusted for age, sex, and BMI; Figure S3: Association of habitual cheese intake with markers of GI inflammation after adjustment for sex, age, BMI; Table S4: Results from linear regression between yogurt intake (cup equivalents per day) from FFQ and markers of GI inflammation adjusted for age, sex, and BMI; Figure S4: Association of habitual yogurt intake with markers of GI inflammation after adjustment for sex, age, BMI.
