# Supplementary material for: No Associations between Dairy Intake and Markers of Gastrointestinal Inflammation in Healthy Adult Cohort
Source: Nutrients. 2023 Aug 8;15(16):3504. doi: 10.3390/nu15163504 (PMC10459578; doi:10.3390/nu15163504)
Supplement: Supplementary file 1 [file nutrients-15-03504-s001.zip › FL100DairyGIInflammation_Supplementary.pdf]

Table S1. Results from linear regression between total dairy intake (cup equivalents per day) from food frequency questionnaire (FFQ) and markers of GI inflammation adjusted for age, sex, and BMI. Results from linear regression between age, sex, or BMI vs. GI inflammation markers are also shown.

	**Transformed Calprotectin**		**Transformed Myeloperoxidase**		**Transformed Neopterin**		**Transformed LPS-binding protein**	
Predictors	*Estimates (95% CI)*	*P-value*	*Estimates (95% CI)*	*P-value*	*Estimates (95% CI)*	*P-value*	*Estimates (95% CI)*	*P-value*
Total Dairy	0.03 (-0.07 – 0.14)	0.544	0.00 (-0.12 – 0.13)	0.949	-0.04 (-0.14 – 0.07)	0.478	0.01 (-0.04 – 0.05)	0.734
Age	-0.00 (-0.01 – 0.00)	0.225	0.00 (-0.01 – 0.01)	0.902	-0.00 (-0.01 – 0.00)	0.314	0.00 (-0.00 – 0.01)	0.216
Sex	0.16 (-0.06 – 0.38)	0.160	0.14 (-0.13 – 0.40)	0.304	0.34 (0.10 – 0.57)	**0.005**	0.15 (0.05 – 0.24)	**0.002**
BMI	0.00 (-0.02 – 0.02)	0.868	-0.00 (-0.03 – 0.02)	0.903	0.02 (-0.01 – 0.04)	0.174	0.04 (0.03 – 0.05)	**<0.001**
R^2 / R^2 adjusted	0.012 / -0.002		0.004 / -0.010		0.043 / 0.030		0.224 / 0.215	

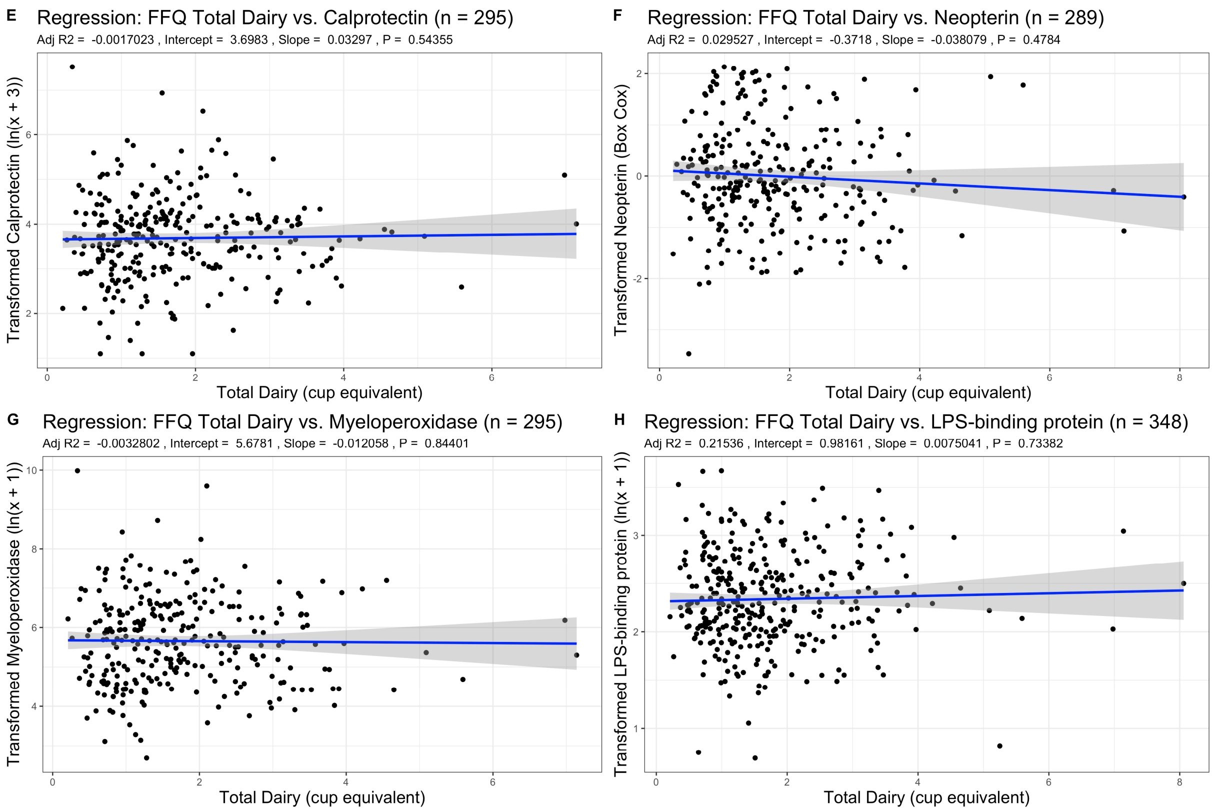

Figure S1. Association of habitual total dairy intake with markers of GI inflammation adjusted for sex, age, BMI.

Table S2. Results from linear regression between fluid milk intake (cup equivalents per day) from FFQ and markers of Gl inflammation adjusted for age, sex, and BMI. Results from linear regression between age, sex, or BMI vs. GI inflammation markers are also shown.

	**Transformed Calprotectin**		**Transformed Myeloperoxidase**		**Transformed Neopterin**		**Transformed LPS-binding protein**	
Predictors	*Estimates (95% CI)*	*P-value*	*Estimates (95% CI)*	*P-value*	*Estimates (95% CI)*	*P-value*	*Estimates (95% CI)*	*P-value*
Fluid Milk	0.03 (-0.14 – 0.19)	0.760	-0.01 (-0.21 – 0.19)	0.924	-0.10 (-0.26 – 0.06)	0.225	-0.01 (-0.08 – 0.05)	0.708
Age	-0.00 (-0.01 – 0.00)	0.229	0.00 (-0.01 – 0.01)	0.905	-0.00 (-0.01 – 0.00)	0.294	0.00 (-0.00 – 0.01)	0.219
Sex	0.15 (-0.07 – 0.37)	0.183	0.13 (-0.13 – 0.40)	0.317	0.33 (0.09 – 0.56)	**0.006**	0.14 (0.04 – 0.23)	**0.004**
BMI	0.00 (-0.02 – 0.02)	0.819	-0.00 (-0.03 – 0.02)	0.915	0.02 (-0.01 – 0.04)	0.169	0.04 (0.03 – 0.05)	**<0.001**
R^2 / R^2 adjusted	0.011 / -0.003		0.004 / -0.010		0.046 / 0.033		0.224 / 0.215	

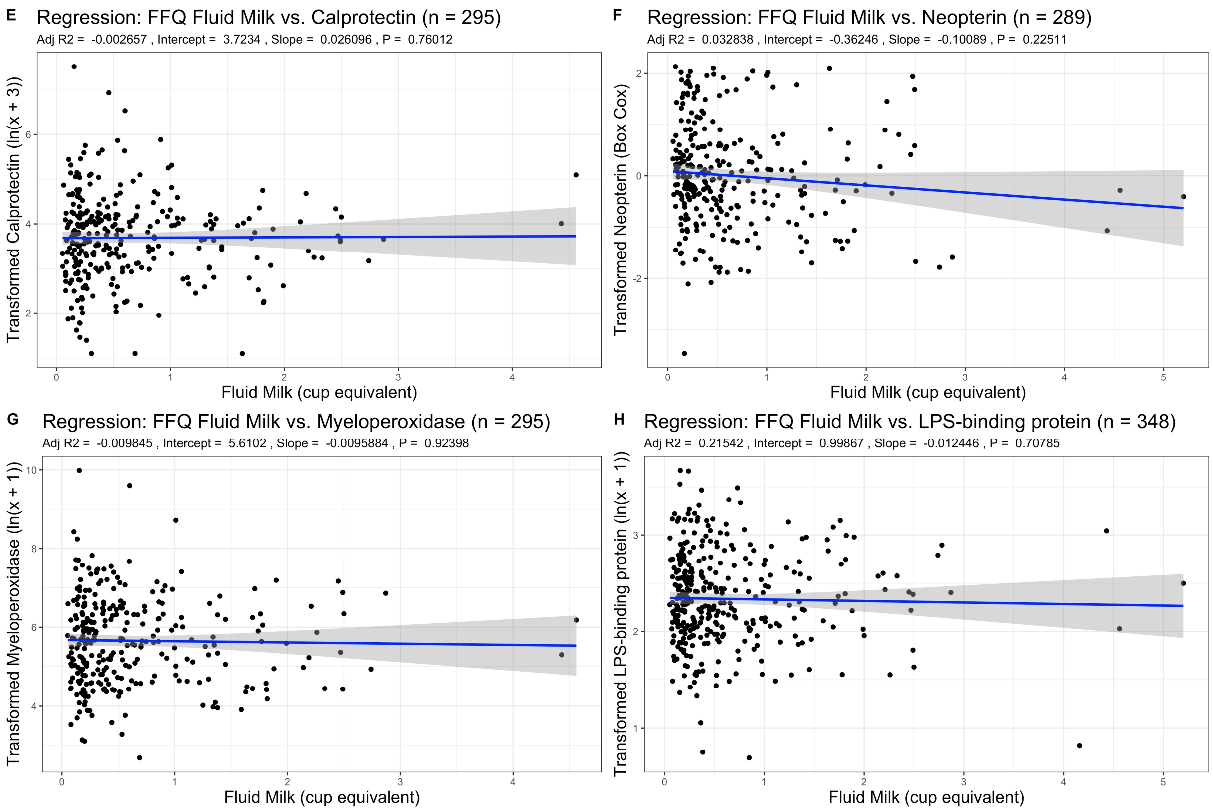

Figure S2. Association of habitual fluid milk intake with markers of GI inflammation after adjustment for sex, age, BMI.

Table S3. Results from linear regression between cheese intake (cup equivalents per day) from FFQ and markers of Gl inflammation adjusted for age, sex, and BMI. Results from linear regression between age, sex, or BMI vs. GI inflammation markers are also shown.

	**Transformed Calprotectin**		**Transformed Myeloperoxidase**		**Transformed Neopterin**		**Transformed LPS-binding protein**	
Predictors	*Estimates (95% CI)*	*P-value*	*Estimates (95% CI)*	*P-value*	*Estimates (95% CI)*	*P-value*	*Estimates (95% CI)*	*P-value*
Cheese	0.05 (-0.14 – 0.24)	0.614	-0.01 (-0.23 – 0.22)	0.963	0.02 (-0.18 – 0.22)	0.846	0.06 (-0.02 – 0.14)	0.171
Age	-0.00 (-0.01 – 0.00)	0.225	0.00 (-0.01 – 0.01)	0.902	-0.00 (-0.01 – 0.00)	0.319	0.00 (-0.00 – 0.01)	0.210
Sex	0.15 (-0.07 – 0.38)	0.172	0.13 (-0.13 – 0.40)	0.309	0.36 (0.13 – 0.60)	**0.002**	0.15 (0.06 – 0.24)	**0.001**
BMI	0.00 (-0.02 – 0.02)	0.881	-0.00 (-0.03 – 0.03)	0.918	0.01 (-0.01 – 0.04)	0.225	0.04 (0.03 – 0.05)	**<0.001**
R^2 / R^2 adjusted	0.012 / -0.002		0.004 / -0.010		0.041 / 0.028		0.228 / 0.219	

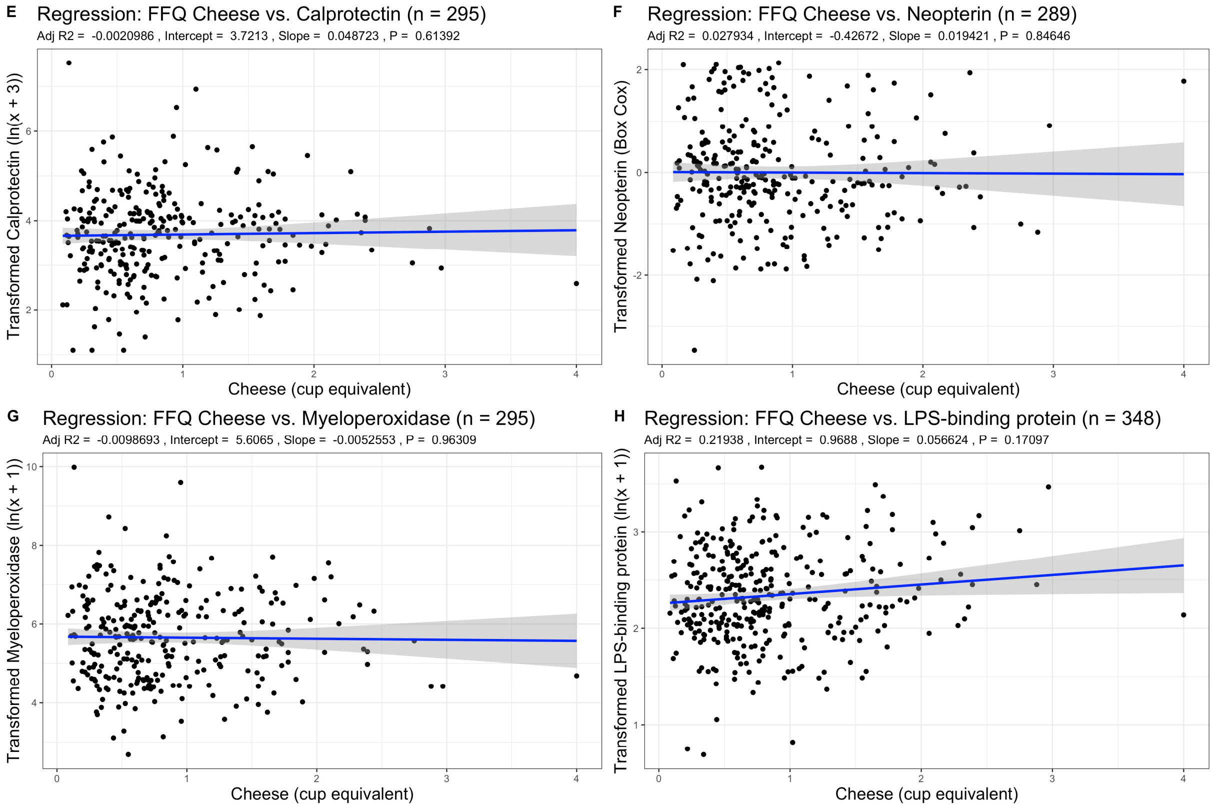

Figure S3. Association of habitual cheese intake with markers of GI inflammation after adjustment for sex, age, BMI.

Table S4. Results from linear regression between yogurt intake (cup equivalents per day) from FFQ and markers of Gl inflammation adjusted for age, sex, and BMI. Results from linear regression between age, sex, or BMI vs. GI inflammation markers are also shown.

	**Transformed Calprotectin**		**Transformed Myeloperoxidase**		**Transformed Neopterin**		**Transformed LPS-binding protein**	
Predictors	*Estimates (95% CI)*	*P-value*	*Estimates (95% CI)*	*P-value*	*Estimates (95% CI)*	*P-value*	*Estimates (95% CI)*	*P-value*
Yogurt	0.08 (-0.38 – 0.54)	0.728	-0.01 (-0.47 – 0.45)	0.955	-0.03 (-0.48 – 0.42)	0.884	-0.04 (-0.23 – 0.14)	0.628
Age	-0.01 (-0.01 – 0.00)	0.148	0.00 (-0.01 – 0.01)	0.944	-0.00 (-0.01 – 0.00)	0.398	0.00 (-0.00 – 0.01)	0.201
Sex	0.16 (-0.07 – 0.39)	0.174	0.13 (-0.11 – 0.36)	0.287	0.35 (0.12 – 0.58)	**0.003**	0.14 (0.05 – 0.23)	**0.002**
BMI	0.00 (-0.02 – 0.02)	0.978	-0.00 (-0.03 – 0.02)	0.812	0.01 (-0.01 – 0.04)	0.207	0.04 (0.03 – 0.05)	**<0.001**
R^2 / R^2 adjusted	0.013 / -0.000		0.004 / -0.010		0.039 / 0.026		0.225 / 0.216	

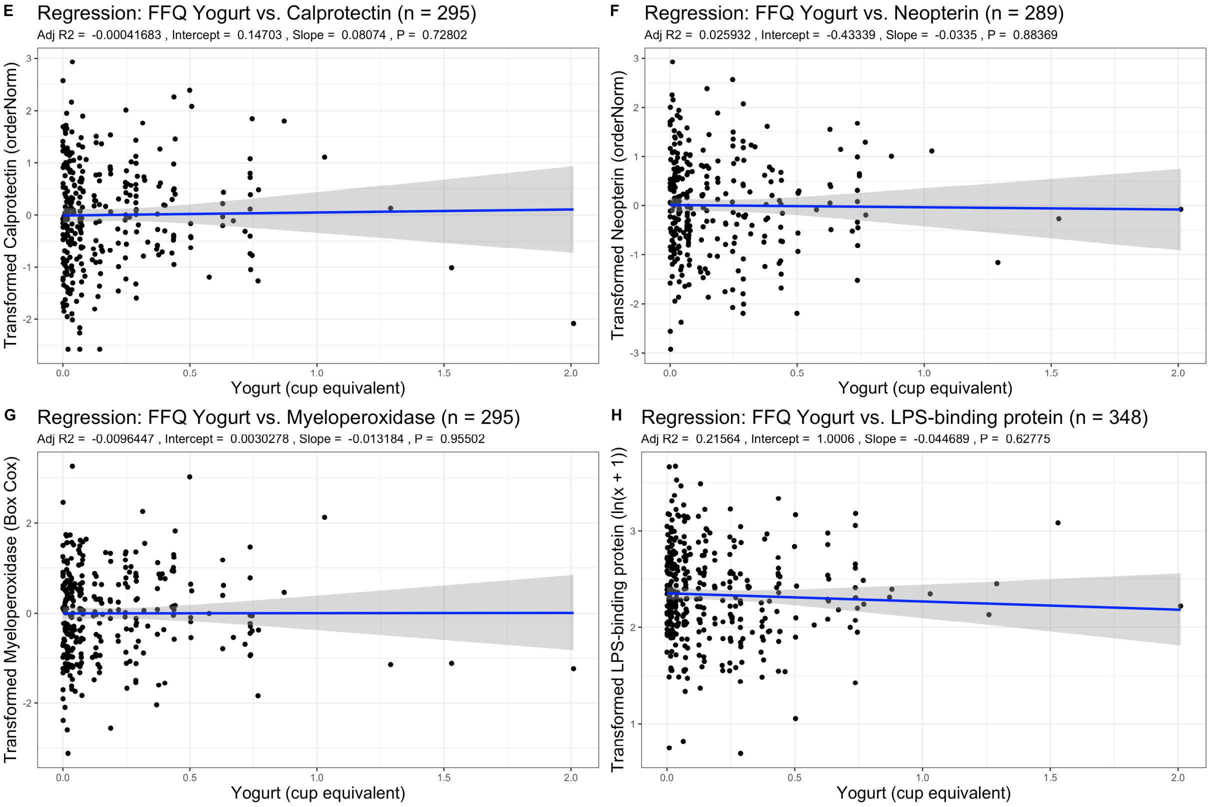

Figure S4. Association of habitual yogurt intake with markers of GI inflammation after adjustment for sex, age, BMI.
